# Large-scale head-to-head comparison of on- and off-target profiles of [^18^F]Tauvid and [^18^F]MK6240 tau-PET in aging and Alzheimer’s disease

**DOI:** 10.21203/rs.3.rs-9987995/v1

**Published:** 2026-11-01

**Authors:** Cécile Tissot, Hsin-Yeh Tsai, Ji Yeon Lee, Nesrine Rahmouni, Firoza Z. Lussier, Corrina S. Fonseca, Joseph Therriault, Jenna Stevenson, Arthur C. Macedo, Mahnaz Shekari, Guilherme Povala, Pâmela C. Lukasewicz Ferreira, Bruna Bellaver, Guilherme Bauer-Negrini, Renaud LaJoie, Brian A. Gordon, Belen Pascual, Val Lowe, David Soleimani-Meigooni, Hwamee Oh, Juan Fortea, William Klunk, Pedro Rosa-Neto, William J. Jagust, Tharick Pascoal, Suzanne L. Baker, HEAD study

**Affiliations:** Lawrence Berkeley National Laboratory; Lawrence Berkeley National Laboratory; Lawrence Berkeley National Laboratory; McGill University; University of Pittsburgh; University of California Berkeley; McGill University; McGill Center for studies in Aging; McGill University; University of California Berkeley; University of Pittsburgh; University of Pittsburgh; University of Pittsburgh; University of Pittsburgh; University of California San Francisco; Washington University in St. Louis; Houston Methodist Research Institute; Mayo Clinic; University of California San Francisco; Brown University; Sant Pau research institute; University of Pittsburgh; McGill University; University of California Berkeley; University of Pittsburgh; Lawrence Berkeley National Laboratory; University of Pittsburgh

**Keywords:** tau-PET, Alzheimer's disease, harmonization

## Abstract

**Background:**

Tau-PET imaging enables *in vivo* quantification of neurofibrillary tangles, but tracers such as [^18^F]Tauvid and [^18^F]MK6240 present distinct affinities and off-target binding properties. Using the multicenter HEAD research cohort, a large-scale study designed to characterize the aging and Alzheimer’s disease (AD) spectrum, this study directly compares tracer performance to support harmonization across cohorts and clinical trials.

**Methods:**

681 participants underwent MRI, amyloid-β (Aβ)-PET, and tau-PET scans with both [^18^F]Tauvid and [^18^F]MK6240. We analyzed regions reflecting AD-related tau deposition (on-target) and known age-related or off-target binding. Analyses included paired t-tests, linear regressions, effect sizes and correlation matrices between the two tracers, across on-target and off-target regions. We further assessed tracer signal separation quantifying the noise to dynamic range ratio (NRR), where lower values indicate better assessment of tau.

**Results:**

Tracers showed strong correlations in on-target regions (R = 0.81–0.95 in the whole cohort; R = 0.83–0.95 in Aβ+; R = 0.59–0.71 in Aβ−), supporting robust tau pathology detection. While off-target regions also correlated (R = 0.58–0.74 in the whole cohort; R = 0.55–0.80 in Aβ+; R = 0.57–0.73 in Aβ−), retention magnitudes differed: [^18^F]Tauvid presented higher retention in the white matter, basal ganglia and choroid plexus, while [^18^F]MK6240 had higher retention in the meninges and extracortical region near the entorhinal cortex. Voxelwise paired t-tests and NRR analyses, conducted at both ROI and voxelwise levels, demonstrated that [^18^F]MK6240 consistently yielded lower NRR values across the cortex, indicating better signal separation, and potentially greater sensitivity for detecting early tau accumulation.

**Conclusion:**

[^18^F]Tauvid and [^18^F]MK6240 capture largely overlapping patterns of tau pathology, but differ notably in the magnitude of off-target binding. These findings highlight the importance of understanding tracer similarities and differences to guide tracer selection and to improve harmonization across studies. Independent and tracer-specific optimizations of pipelines are essential before harmonization across tracers can be achieved, thereby facilitating accurate cross-study comparisons and improving clinical translation.

**Trial Registration:**

NCT05361382

## Introduction

Tau neurofibrillary tangles, a hallmark of Alzheimer’s disease (AD), can be quantified *in vivo* via positron emission tomography (PET). However, different tau-PET tracers exhibit distinct off-target retention patterns that complicate signal quantification. [^18^F]Tauvid, [^18^F]THK5117, [^18^F]THK5317, [^18^F]THK5351, and [^11^C]PBB3 are associated with MAO-B, α-synuclein, and choroid plexus signal (1-4), while [^18^F]MK6240, [^18^F]RO948, [^18^F]PI2620 and [^18^F]GTP1 demonstrate age-related and extracerebral binding (5-9).

As tau-PET is increasingly used for disease staging and clinical trial stratification (10), understanding tracer-specific differences is essential for harmonization and cross-study comparison. To achieve such harmonization, it is crucial to assess how the spatial characteristics of tracer binding varies, particularly the relationship between on-target and off-target regions. Accordingly, tracer selection should reflect the intended application, as differences in off-target retention, sensitivity, and signal characteristics may affect their suitability for staging, cross-sectional analyses, or longitudinal monitoring. However, prior head-to-head studies have been limited by small sample sizes, restricting comprehensive evaluation of tracer-specific signal properties and regional binding patterns (11). Specifically, small studies lacked the power to reliably identify and characterize tracer performance within the critical early accumulation ranges, which is the window where subtle tau changes, and the impact of potential interventions are hypothesized to be most pronounced (12, 13).

To address this gap, the HEAD study (**H**ead-to-head **E**valuation of tau tracers in **A**lzheimer’s **D**isease) evaluated individuals along the aging and AD spectrum using [^18^F]Tauvid and [^18^F]MK6240 tau-PET, amyloid-β (Aβ) PET and MRI (Clinical trial: NCT05361382; registration date: 2022-04-29). While the initial HEAD manuscript (14) established diagnostic thresholds and rates of positivity based on Aβ status, the underlying relationships between on- and off-target binding, and how these dynamics influence quantification, remain poorly understood. Therefore, we sought to quantify the associations between on-target and off-target regions for [^18^F]Tauvid and [^18^F]MK6240 to establish a foundational understanding of their distinct binding profiles and to guide future harmonization efforts. Using data from 681 subjects, we aimed to: 1) assess associations of on-target and off-target regions between [^18^F]Tauvid and [^18^F]MK6240, 2) evaluate cross-regional associations between tracers, and 3) compare their signal characteristics, including dynamic range and noise between target and off-target regions.

## Methods

### Dataset:

Data were obtained from 681 participants in the multi-site HEAD study (USA, Canada, and Spain; see Supplementary Material). The project was approved by Advarra Institutional Review Board in the USA, the Douglas Research Institute Research Ethics Board in Canada, and the ethics committee of the Fundació de Gestió Sanitària Hospital de la Santa Creu i Sant Pau in Spain, in accordance with the Declaration of Helsinki. All participants provided written informed consent. All underwent MRI, [^18^F]Tauvid and [^18^F]MK6240 tau-PET, Aβ-PET, and neuropsychological assessment.

Participants were classified as cognitively unimpaired (CU) young (CUY) [≤ 25 years of age], CU older adults (CUO) [≥ 50 years of age], mild cognitive impairment (MCI), or Alzheimer’s disease (AD) based on clinical evaluation. Aβ-negative cognitively impaired (CI) individuals were grouped separately, which demographic information can be found in Supplementary Table 1. Further details are provided in the Supplementary Material.

### PET processing:

[^18^F]Tauvid and [^18^F]MK6240 PET scans were acquired 80–100 and 90–110 minutes post-injection, respectively (15), and processed using harmonized ADNI-based reconstruction parameters. Images were realigned, averaged, and coregistered to T1-weighted MRI. SUVRs were calculated in native space using inferior cerebellar grey as the reference region (6, 16) within Berkeley PET imaging pipeline (17), and smoothed to 6 mm^3^ FWHM. Regions of interest, including Braak I–VI, meta-temporal, whole cortex, and CenTauR masks, were defined in native space using FreeSurfer (18-20). Off-target regions included pallidum, putamen, caudate, thalamus, choroid plexus, meninges, eroded white matter and the extracortical hotspot medial to the entorhinal cortex (EntoEch) (6, 7). Aβ status was determined by consensus visual reads. Further details are provided in the Supplementary Material.

### Statistical analyses:

Statistical analyses were conducted in R (version 2023.09) and MATLAB (version 2024b). Mean and standard deviation PET images were generated stratified by both diagnostic group and Aβ status. We compared demographic characteristics across diagnostic groups, using analysis of variance (ANOVA) for continuous variables and chi-square tests for categorical variables. Voxelwise paired t-tests and linear models between tracers were performed using VoxelStats (21) to identify spatial patterns of differences in tracer uptake. To assess associations between tracers, we implemented linear regression models and calculated Spearman correlation matrices between [^18^F]Tauvid and [^18^F]MK6240 regional values, within the whole cohort and stratified by Aβ status, using both SUV and SUVR values. These analyses were performed separately in on-target and off-target regions. We further examined regional relationships by generating correlation matrices across ROIs, both within and across tracers.

To assess how effectively tracers capture tau signal, we evaluated a noise-to-range ratio (NRR), defined as the standard deviation of noise divided by that across subjects (i.e., the dynamic range): $\text{NRR}=\frac{Standard\;deviation\;of\;noise}{Standard\;deviation\;of\;across\;many\;subjects\;(range)}$. This metric allows for the quantification of signal separation. NRR was measured at both the region-of-interest level for on-target regions, and at the voxel-wise level. The noise group comprised CUY individuals, while the dynamic range included all diagnostic groups.

## Results

We assessed 681 individuals along the aging and AD spectrum. There was no age difference between older groups. There was a higher proportion of females in CUY and CUO Aβ− than males, and higher proportion of males in CI Aβ−. [^18^F]Tauvid and [^18^F]MK6240 meta-temporal ROIs showed significant differences across diagnostic groups, with higher values for groups indicating cognitive impairment and Aβ pathology (Table 1).

### Visual differences between tracers:

Mean images revealed distinct tracer profiles (Fig. 1A). In CUY, [^18^F]Tauvid showed higher brain-wide signal than [^18^F]MK6240, while both exhibited extracortical binding near the entorhinal cortex. In CUO Aβ−, [^18^F]Tauvid remained higher in white matter and subcortical regions, while [^18^F]MK6240 was higher in the extracortical hotspot. Conversely, CUO Aβ + individuals showed higher [^18^F]MK6240 retention in temporal regions. Overall, [^18^F]Tauvid demonstrated higher apparent uptake at low tau burden, whereas [^18^F]MK6240 exceeded [^18^F]Tauvid at higher binding levels, suggesting [^18^F]Tauvid may produce more widespread low-level signal in regions not expected to accumulate tau early, although it remains unclear whether this represents early tau or non-specific off-target binding. In MCI Aβ + and AD Aβ+, both tracers showed similar spatial patterns, with greater [^18^F]MK6240 SUVR magnitude. In CI Aβ−, [^18^F]Tauvid binding was higher throughout the brain, particularly in white matter. Although [^18^F]MK6240 exhibited meningeal spill-in in non-AD groups, uptake in on-target regions in MCI/AD Aβ + exceeded this contribution, indicating predominance of disease-related signal. Standard deviation images demonstrated high temporal lobe variability for both tracers in Aβ + groups, with greater magnitude for [^18^F]MK6240, alongside high meningeal variability (Supplementary Fig. 1).

Binarized voxelwise paired t-tests (Fig. 1B) showed [^18^F]MK6240 had higher meningeal uptake, whereas [^18^F]Tauvid was higher in white matter. In CUY and CUO, higher [^18^F]Tauvid signal was observed outside the skull and across the whole cortex. Despite elevated entorhinal uptake on mean images, [^18^F]MK6240 was not significantly higher than [^18^F]Tauvid in this region within our range. With increasing Aβ burden, the extent of [^18^F]MK6240-dominant voxels expanded, reflecting hierarchical tau spread, from temporal to parietal and frontal regions. CI Aβ− patterns resembled those of CUY and CUO Aβ−.

Linear models quantified the relationship between [^18^F]Tauvid and [^18^F]MK6240 across diagnostic groups and Aβ statuses (Fig. 1C). The visualization reveals distinct patterns of correlation. First, we observed high positive t-values in MCI and AD Aβ + individuals, showing a strong correlation between the tracers, especially in AD-related regions. This confirms the high on-target correlation observed in the full cohort for individuals with established AD pathology. In the Aβ− groups (CUY and CUO Aβ−), the correlation strength was generally lower across the brain, consistent with the lower on-target SUVRs observed in these groups. Interestingly, we observed strong associations in CUO Aβ− around meningeal, as well as white matter regions. Throughout the diagnoses, we observed a positive association in subcortical nucleus uptake, except in CUY. The map clearly delineates where the relationship is strongest, underscoring that the relationship between [^18^F]Tauvid and [^18^F]MK6240 is driven by the presence of established AD pathology rather than non-specific binding.

### Associations in on-target regions:

We assessed associations between [^18^F]Tauvid and [^18^F]MK6240 in Braak ROIs, meta-temporal, and whole cortex in the full cohort and stratified by Aβ status. All linear models were statistically significant. Full-cohort correlations were high [Braak I: R = 0.92, II: 0.81, III: 0.94, IV: 0.95, V: 0.94, VI: 0.87; meta-temporal: 0.95; whole cortex: 0.94]. In Aβ− individuals, correlations ranged from 0.59 to 0.71 [Braak I: 0.71, II: 0.68, III: 0.62, IV: 0.61, V: 0.59, VI: 0.63; meta-temporal: 0.64; whole cortex: 0.59], whereas in Aβ + values were higher (R = 0.83–0.95) [Braak I: 0.89, II: 0.83, III: 0.94, IV: 0.95, V: 0.95, VI: 0.91; meta-temporal: 0.95; whole cortex: 0.94] (Fig. 2A; histograms can be found in Supplementary Fig. 2, and information on intercepts and slopes in Supplementary Table 2).

SUVRs were lowest in CUY individuals in early Braak regions (I-III) and increased progressively with Aβ status and cognitive impairment, except for [^18^F]Tauvid in Braak II where CUO Aβ− and CUO Aβ + were similar. CI Aβ− group resembled CUO Aβ−. Diagnostic-group comparisons showed substantial effect sizes for both tracers across many ROIs. In early Braak regions (I–III), effect sizes for the CUO Aβ− versus CUO Aβ + comparison ranged from 0.49 to 0.96 for [^18^F]Tauvid and from 0.97 to 1.22 for [^18^F]MK6240, with the largest difference between tracers in Braak II. The strongest effects were generally observed for comparisons between CUO Aβ− and AD Aβ+, with Cohen’s d ranging from 1.87 to 3.51 for [^18^F]Tauvid and 2.06 to 3.95 for [^18^F]MK6240 across ROIs. Effect sizes were often larger for [^18^F]MK6240 in comparisons spanning greater tau burden, although their relative magnitude varied by diagnostic contrast and ROI (Supplementary Fig. 3). Consistent with this pattern, [^18^F]MK6240 exhibited a broader dynamic range in individuals with high Aβ burden and cognitive impairment (Fig. 2B). Partial volume corrected SUVR analyses can be found in Supplementary Fig. 4.

CenTauR masks were additionally used to provide a tracer-agnostic regional framework. Analyses showed similarly strong correlations in the whole cohort (R = 0.92–0.95), higher in Aβ+ (R = 0.90–0.95) than Aβ− (R = 0.56–0.69) (Supplementary Fig. 5).

To evaluate whether between-tracer differences were influenced by reference-region normalization, we additionally examined SUV values in the inferior cerebellar grey, and selected on-target ROIs. Inferior cerebellar grey SUV was moderately correlated between [^18^F]Tauvid and [^18^F]MK6240 (R = 0.65) with similar correlations in Aβ− (R = 0.60) and Aβ+ (R = 0.70) individuals. On-target ROI SUVs were also positively correlated between tracers, with whole-cohort correlations of R = 0.85 in Braak I, R = 0.72 in Braak II, R = 0.80 in Braak III/IV, R = 0.74 in Braak V/VI, and R = 0.84 in the meta-temporal region. Corresponding correlations were R = 0.62–0.74 in Aβ− individuals and R = 0.73–0.86 in Aβ + individuals (Supplementary Fig. 6).

### Associations in age-related and off-target regions:

In the whole cohort, associations were strong [pallidum: R = 0.70, putamen: 0.69, caudate: 0.65, thalamus: 0.63, choroid plexus: 0.58, EntoEch: 0.58, meninges: 0.65, white matter: 0.74]. Correlations were generally higher in Aβ− than Aβ + individuals. In Aβ−, values ranged from 0.57 to 0.73 [pallidum: R = 0.73, putamen: 0.73, caudate: 0.71, thalamus: 0.65, choroid plexus: 0.61, EntoEch: 0.57, meninges: 0.65, white matter: 0.61]. In Aβ+, correlations ranged from 0.55 to 0.80 [pallidum: R = 0.61, putamen: 0.64, caudate: 0.60, thalamus: 0.58, choroid plexus: 0.55, EntoEch: 0.60, meninges: 0.66, white matter: 0.80] (Fig. 3A; histograms can be found in Supplementary Fig. 7 and information on intercepts and slopes in Supplementary Table 3).

[^18^F]Tauvid exhibited higher SUVRs in subcortical nuclei (pallidum, putamen, caudate, thalamus), choroid plexus, and white matter, whereas [^18^F]MK6240 was higher in EntoEch and meninges across all subjects. CUY individuals showed lower SUVRs in subcortical regions, with higher [^18^F]Tauvid values. While choroid plexus values were similar across groups, [^18^F]Tauvid remained higher; conversely, [^18^F]MK6240 showed higher EntoEch and meningeal magnitudes. White matter exhibited greater variability for [^18^F]MK6240, particularly in AD Aβ+, whereas [^18^F]Tauvid values were more consistent, likely reflecting partial volume effects (Fig. 3B; Supplementary Fig. 8). Mean (SD) SUVR in the meninges was 1.25 (0.37) in Aβ− and 1.22 (0.32) in Aβ + for [^18^F]MK6240 versus 0.81 (0.26) and 0.76 (0.19) for [^18^F]Tauvid, and in the white matter was 0.79 (0.15) and 1.01 (0.34) for [^18^F]MK6240 versus 1.18 (0.13) and 1.27 (0.17) for [^18^F]Tauvid. Despite the greater dynamic range observed with [^18^F]MK6240, it is believed that the prominent meningeal uptake could obscure the full extent of this dynamic range at the individual-subject level. In the full cohort (N = 681), meta-temporal uptake exceeded the subject-specific meningeal signal in 327 participants (48.0%). In comparison, meta-temporal uptake exceeded subject-specific white-matter uptake in 400 participants (58.7%) for [^18^F]Tauvid. Thus, although [^18^F]MK6240 demonstrated greater dynamic range, its variable meningeal signal frequently approached or exceeded meta-temporal uptake, whereas [^18^F]Tauvid showed a more consistent separation between meta-temporal and white-matter uptake.

### Associations within and between tracers in on-target and off-target regions:

Correlation matrices assessed relationships between off-target regions and selected on-target ROIs, stratified by Aβ status (Fig. 4; p < 0.05 shown). On-target regions were positively correlated within tracer in both groups, with stronger associations between neighboring Braak ROIs [AβȒ: [^18^F]Tauvid R = 0.49 (Braak I vs V-VI) to 0.85 (III-IV vs V-VI), [^18^F]MK6240 R = 0.41 (I vs V-VI) to 0.84 (II vs III-IV); Aβ+: [^18^F]Tauvid R = 0.55 (II vs V-VI) to 0.92 (III-IV to V-VI), [^18^F]MK6240 R = 0.68 (I vs V-VI) to 0.94 (I vs II)].

In Aβ−, pallidum and thalamus showed strong similar correlations with Braak ROIs, for both tracers, particularly Braak II-thalamus ([^18^F]Tauvid: R = 0.80, [^18^F]MK6240: R = 0.81), which decreased in Aβ+, more markedly for [^18^F]Tauvid. Choroid plexus was associated with all Braak ROIs in Aβ−, strongest for Braak II due to its proximity ([^18^F]Tauvid: R = 0.74, [^18^F]MK6240: R = 0.70) and remained higher for Braak II in Aβ + despite lower values ([^18^F]Tauvid: R = 0.34, [^18^F]MK6240: R = 0.39). Meningeal binding differed by tracer: in Aβ− [^18^F]Tauvid Braak III-IV was negatively correlated (R=−0.15), whereas [^18^F]MK6240 showed positive correlations increasing with stage (Braak III–IV: R = 0.16; V–VI: R = 0.36). In Aβ+, both tracers showed similar positive associations ([^18^F]Tauvid: R = 0.18 Braak III-IV, 0.20 Braak V-VI; [^18^F]MK6240: R = 0.21 Braak III-IV, 0.20 V-VI). EntoEch was positively associated with Braak I in Aβ− ([^18^F]Tauvid: R = 0.17; [^18^F]MK6240: R = 0.33); in Aβ+, this association disappeared for [^18^F]Tauvid and decreased for [^18^F]MK6240 (R = 0.20). White matter depicted high correlations with Braak ROIs in both groups, increasing in Aβ+ ([^18^F]Tauvid: Aβ− R = 0.42–0.85, Aβ + R = 0.68–0.92; [^18^F]MK6240: Aβ− R = 0.56–0.86, Aβ + R = 0.72–0.97), likely due to cortical spill-in. Interestingly, pallidum and thalamus exhibited strong correlations with white matter signal, that were markedly attenuated in Aβ + . Off-target correlations between EntoEch and meninges remained high regardless of tracer or Aβ status ([^18^F]Tauvid: Aβ− R = 0.49, Aβ + R = 0.42; [^18^F]MK6240: Aβ− R = 0.49, Aβ + R = 0.50).

Across tracers, on-target agreement was higher in Aβ + than Aβ−, while several off-target regions displayed the opposite pattern, suggesting more divergent tracer behavior in Aβ + . Among regions prone to partial volume effects (meninges, EntoEch, WM), only WM increased in Aβ+, likely reflecting spill-in from adjacent on-target uptake.

When focusing more specifically in off-target regions, we observed that retention magnitudes differed significantly between the two tracers (paired t-tests, *p* < 0.05). [^18^F]MK6240 showed higher SUVR in the meninges and EntoEch, whereas [^18^F]Tauvid exhibited higher retention in the white matter, pallidum, putamen, caudate, thalamus and choroid plexus (Supplementary Fig. 9), in both Aβ− and Aβ + groups.

Further exploratory analyses were conducted by including age and/or sex as covariates in the models; however, the inclusion of these variables did not significantly improve model performance.

### Noise-to-dynamic range ratio of tau-PET tracers:

Noise-to-dynamic range ratio (NRR) analysis across Braak ROIs, meta-temporal ROI, and whole cortex demonstrated that [^18^F]MK6240 consistently yielded lower NRR values than [^18^F]Tauvid (Fig. 5A), indicating superior tau assessment. In early Braak ROIs, [^18^F]Tauvid NRR was 0.33 (Braak I) and 0.62 (Braak II), compared to 0.19 and 0.23 for [^18^F]MK6240, respectively. Intermediate and later stages showed similar patterns, with [^18^F]Tauvid values of 0.34 (Braak III), 0.30 (Braak IV), 0.35 (Braak V), 0.71 (Braak VI) versus 0.15, 0.15, 0.17 and 0.33 for [^18^F]MK6240. Meta-temporal ROI yielded the lowest NRR values across all ROIs, with [^18^F]MK6240 being more favorable (0.12 vs. 0.23), and whole-cortex NRR was 0.18 ([^18^F]MK6240) vs. 0.38 ([^18^F]Tauvid). This corresponded to reductions in NRR of 44.1% (Braak I), 63.4% (Braak II), 56.0% (Braak III), 51.5% (Braak IV), 50.9% (Braak V), and 53.1% (Braak VI), 49.2% (meta-temporal) and 52.9% (whole cortex) (Supplementary Table 4). Standard deviation values used to calculate NRR can be found in Supplementary Fig. 10.

Voxel-wise analyses corroborated these results, showing lower cortical NRR for [18F]MK6240, particularly in AD-affected regions, while off-target NRR values remained similar between tracers (Fig. 5B).

Results were comparable in a sub-analysis of CUO Aβ− individuals < 65 years (Supplementary Fig. 10&11; Supplementary Table 5).

## Discussion

Our multicenter head-to-head comparison reveals that while [^18^F]Tauvid and [^18^F]MK6240 share high on-target correlation, they are distinguished by divergent off-target profiles and signal-to-noise characteristics. Supporting their validity to detect tau pathology, both tracers showed similar patterns of tau-PET uptake in regions expected to accumulate tau, with high correlations across on-target regions, driven by Aβ + individuals. The variability noted within Aβ− individuals was most likely due to tracer-specific differences in off-target regions, impacting on-target quantification. To further clarify how off-target retention influences on-target quantification, we assessed each tracer’s NRR by examining the interplay between its dynamic range and noise, particularly the inter-subject cortical variability introduced by off-target signal in participants believed to have no tau. These analyses highlighted [^18^F]MK6240 provided superior quantitative assessment of tau, with consistently lower NRR values at the ROI-wise and voxel-wise levels, driven by its higher dynamic range and similar noise signal to [^18^F]Tauvid. This superior NRR suggests [^18^F]MK6240 may offer enhanced sensitivity for detecting subtle tau accumulation in early-stage disease or during pharmaceutical interventions.

[^18^F]Tauvid and [^18^F]MK6240 consistently showed high correlations in regions where we expect tau accumulation, with stronger correlations in individuals already showing AD-related pathology. Both tracers can capture the incremental tau binding increase associated with Aβ positivity and cognitive impairment (11, 22, 23). When examining inter-tracer correlations, at both the ROI and voxel-wise levels, we found that associations were generally stronger in Aβ + . In contrast, Aβ− showed comparatively higher correlations involving off-target regions, both with other off-target and on-target regions. The lowest association was observed within Braak II ROI (hippocampus), likely reflecting the known susceptibility of [^18^F]Tauvid to choroid plexus spillover (4), though the maintained high correlation suggests this signal remains clinically interpretable. Nevertheless, strong inter-tracer correlations should not be interpreted as agreement in measured SUVRs, or as evidence that the measurements are interchangeable. Differences in regression slopes by Aβ status indicate that the relationship between tracers changes across the disease continuum. Consequently, a linear harmonization across the full cohort may be less reliable at low tau burden, where inter-tracer associations are weaker and off-target effects may have a greater relative influence.

Our study corroborated previous findings that [^18^F]Tauvid presents off-target binding in the white matter, choroid plexus, and age-related off-target in some subnuclei regions (7, 24). In turn, [^18^F]MK6240 depicted off-target binding in the meninges, an extracortical area close to the entorhinal cortex, and age-related off-target binding similar to that of [^18^F]Tauvid (6, 15, 25). Importantly, we found that off-target signal from one tracer was often highly correlated with those of the other, with differences primarily driven by the uptake magnitude. This suggests the biological underpinnings of off-target binding are similar for both tracers. When comparing between Aβ groups in off-target regions, we observed lower correlations within Aβ + as compared to Aβ−, suggesting that while both tracers capture consistent off-target binding patterns, they differ in signal intensity. When assessing SUVR correlations in on-target regions, all associations were strong, indicating that the measured signal is largely driven by true on-target binding rather than spillover from off-target retention.

Both mean and standard-deviation maps showed higher values and greater between-subject variability for [^18^F]MK6240 in several Aβ + groups, which may reflect greater sensitivity to between-person differences in uptake. [^18^F]Tauvid produces more widespread low-level signal in low-tau groups (CUY and CUO Aβ−), potentially reflecting non-specific binding. Analysis of the SUVs provided additional information regarding the potential effect of reference-region normalization. Target-region SUVs were associated between tracers, whereas inferior cerebellar grey SUVs showed a more moderate association, indicating that differences in reference-region uptake may contribute to the magnitude of some SUVR differences. Reference-region SUV associations were broadly comparable across Aβ groups but these cross-sectional results cannot establish reference-region stability (26). The higher [^18^F]Tauvid reference-region SUV may reflect age-related cerebellar increases reported by arterial input function modeling (27), but comparable [^18^F]MK6240 data is missing. Calculating ROIs in native space reduces anatomical misclassification but does not establish reference-region stability or eliminate potential off-target contamination. On-target correlations were broadly similar after partial-volume correction. Nevertheless, differences in off-target binding profiles represent a plausible contributor to the discrepant signal observed between tracers. Consistently, voxel-wise paired comparisons revealed spatially distinct patterns of higher uptake between tracers, suggesting regional differences may reflect a combination of true on-target binding and non-specific retention. Regarding on-target binding, tracer binding properties such as distinct sensitivity to either 4R or 3R could play a role. Tau tangle maturity studies suggest a predominance of 4R tau at early stages of the disease, while more 3R tau at later ones (28). In turn, in silico modeling predicted that [^18^F]Tauvid binds better to 3R, while [^18^F]MK6240 to 3R/4R tangles (29). [^18^F]MK6240 possibly binds to tau tangles with higher affinity, leading to higher SUVRs in AD-related regions. Further clarification of these mechanisms will require longitudinal data, such as those from the HEAD study.

We assessed whether [^18^F]Tauvid or [^18^F]MK6240 showed higher assessment of tau by calculating the NRR. Using CUY and CUO Aβ− participants under 65 years as noise groups produced comparable results, supporting the robustness of our approach. Using these groups limits expected age-related or early tau contribution. As anticipated, NRR was lowest, reflecting better tau assessment, in regions known to accumulate tau in AD (18). Our findings indicate [^18^F]MK6240 provides higher assessment across all Braak ROIs and global cortical measures, with the largest differences observed in Braak II and VI ROIs. Braak II differences might be due to choroid plexus retention spilling into SUVR calculations (24). Standard deviations in the noise groups were similar between [^18^F]Tauvid and [^18^F]MK6240, whereas [^18^F]MK6240 showed a greater spread of uptake across the full cohort, which is reflected in its larger slope differences between Aβ + and Aβ− groups. Its lower NRR possibly reflects a larger cross-sectional signal range relative to baseline variability. Specifically for Braak VI, ROI differences are likely due to [^18^F]MK6240's broader dynamic range at high tau load, as it might better distinguish advanced pathology. These findings suggest that while both tracers reliably detect tau, NRR measurement reflects the balance between dynamic range and noise. [^18^F]MK6240’s broader dynamic range improves its ability to distinguish tau burden levels cross-sectionally, whereas variability driven by off-target signal introduces noise for both tracers and can hinder low-level signal. Additionally, because NRR uses the full-cohort standard deviation rather than a specific diagnostic contrast, we also assessed effect sizes across diagnoses and ROIs. [^18^F]MK6240 depicted larger effect sizes in early Braak regions (entorhinal cortex and hippocampus) between CUO Aβ− and CUO Aβ+, whereas [^18^F]Tauvid did so in the whole cortex between CUY and CUO Aβ+, illustrating that the relative advantage varies across ROIs and diagnosis comparisons.

Our findings underscore the importance of tracer selection based on the intended use, particularly in the context of clinical trials. Research has aimed at determining specific regions to be used when tracking disease progression (30). Due to its low NRR and high dynamic range, [^18^F]MK6240 might be particularly suited to identify subtle early tau changes (31). We hypothesize that this profile may offer an advantage for tracking disease progression and detect subtle longitudinal differences in pharmaceutical trials, however this requires longitudinal validation. Indeed, [^18^F]MK6240 has substantial and variable meningeal uptake, including in participants with low tau burden (26). Because this signal is adjacent to cortical target regions, spill-in may make subtle regional cortical uptake difficult to interpret in some individuals. In contrast, [^18^F]Tauvid is a more established tracer that, despite choroid plexus off-target binding, is more widely available and has extensive validation, including autopsy validation (32). Furthermore, at the individual level, meta-temporal uptake exceeded subject-specific white-matter uptake in 58.7% of participants for [^18^F]Tauvid, whereas meta-temporal uptake exceeded subject-specific meningeal uptake in 48% for [^18^F]MK6240. Thus, [^18^F]MK6240 meningeal retention more frequently approached or exceeded meta-temporal uptake, potentially complicating interpretation of subtle cortical signal in individual scans. Because different off-target regions are compared, this finding does not establish which tracer measures individual-level tau more accurately. Overall these tracer differences highlight that tracer choice should be guided by the specific research or clinical objective, as variation in off-target binding magnitude and signal characteristics may influence the interpretation of regional uptake, and ultimately impact disease staging, differential diagnosis, and clinical trial enrollment.

This study has several inherent limitations. First, this is a cross-sectional analysis, so longitudinal sensitivity, test–retest reproducibility, and reference-region stability over time could not be assessed. Second, the predominantly White study population may limit generalizability across racially diverse cohorts, where tau kinetics may vary. Third, although multiple scanners were used across study sites, all images underwent a standardized acquisition and processing pipeline, which helps mitigate variability but cannot fully eliminate potential scanner-related differences. Finally, we do not have autopsy data, and there is limited [^18^F]MK6240 validation against post-mortem tau pathology. This limits our ability to confirm that regional PET signal fully reflects true tau deposition and highlights the need for future studies incorporating autopsy measures to validate tracer-specific findings.

Together, these findings emphasize the feasibility but also the challenges of harmonizing tau-PET tracers. While [^18^F]Tauvid and [^18^F]MK6240 capture largely overlapping patterns of tau pathology, their off-target binding maps, though similar in distribution, differ notably in magnitude. These differences underscore that careful selection of the tracer, and independent optimization of processing pipelines for each tracer beforehand, is crucial before attempting harmonization. Doing so can improve the accuracy of longitudinal analyses, enhance sensitivity to early pathology, and reduce potential confounds from off-target signals. Ultimately, understanding both the overlapping and unique properties of [^18^F]Tauvid and [^18^F]MK6240 allows for more precise interpretation of tau-PET data, better design of studies, and informed decision-making in clinical and research contexts.

## Supplementary Material

This is a list of supplementary files associated with this preprint. Click to download.

• HEADTissotSupplMaterialR1.pdf

## Figures and Tables

**Figure 1:**
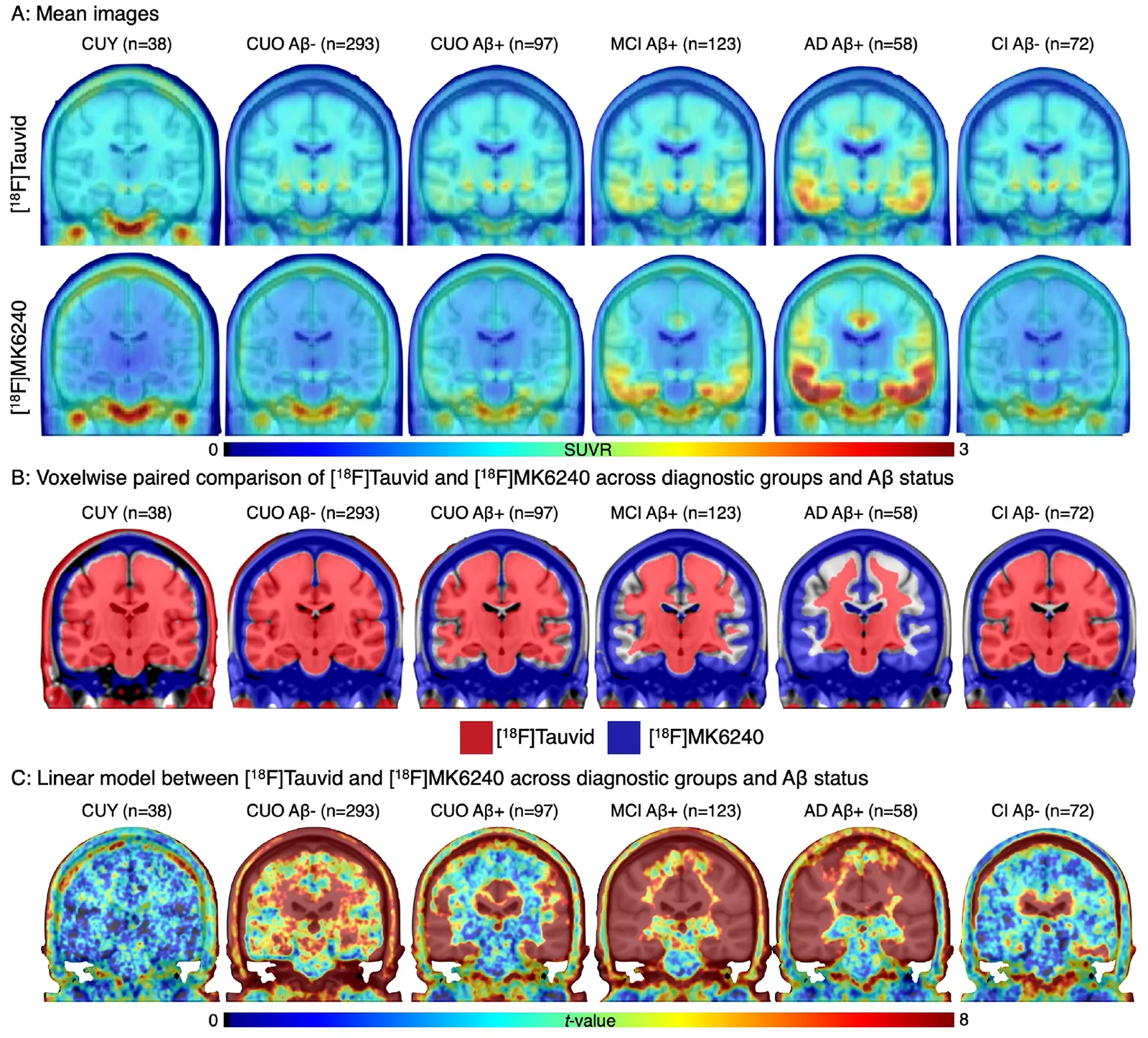
Mean images based on diagnostic group and Aβ status Tau-PET signal and regional positivity across diagnostic and Aβ status groups. A. Mean image of diagnostic group and Aβ status. B. Voxelwise paired t-test of [^18^F]Tauvid and [^18^F]MK6240 across diagnostic groups and Aβ status, indicating the direction of significant differences between tracers. All voxels shown were statistically significant. C: Linear model between [^18^F]Tauvid and [^18^F]MK6240 across diagnostic groups and Aβ status.

**Figure 2:**
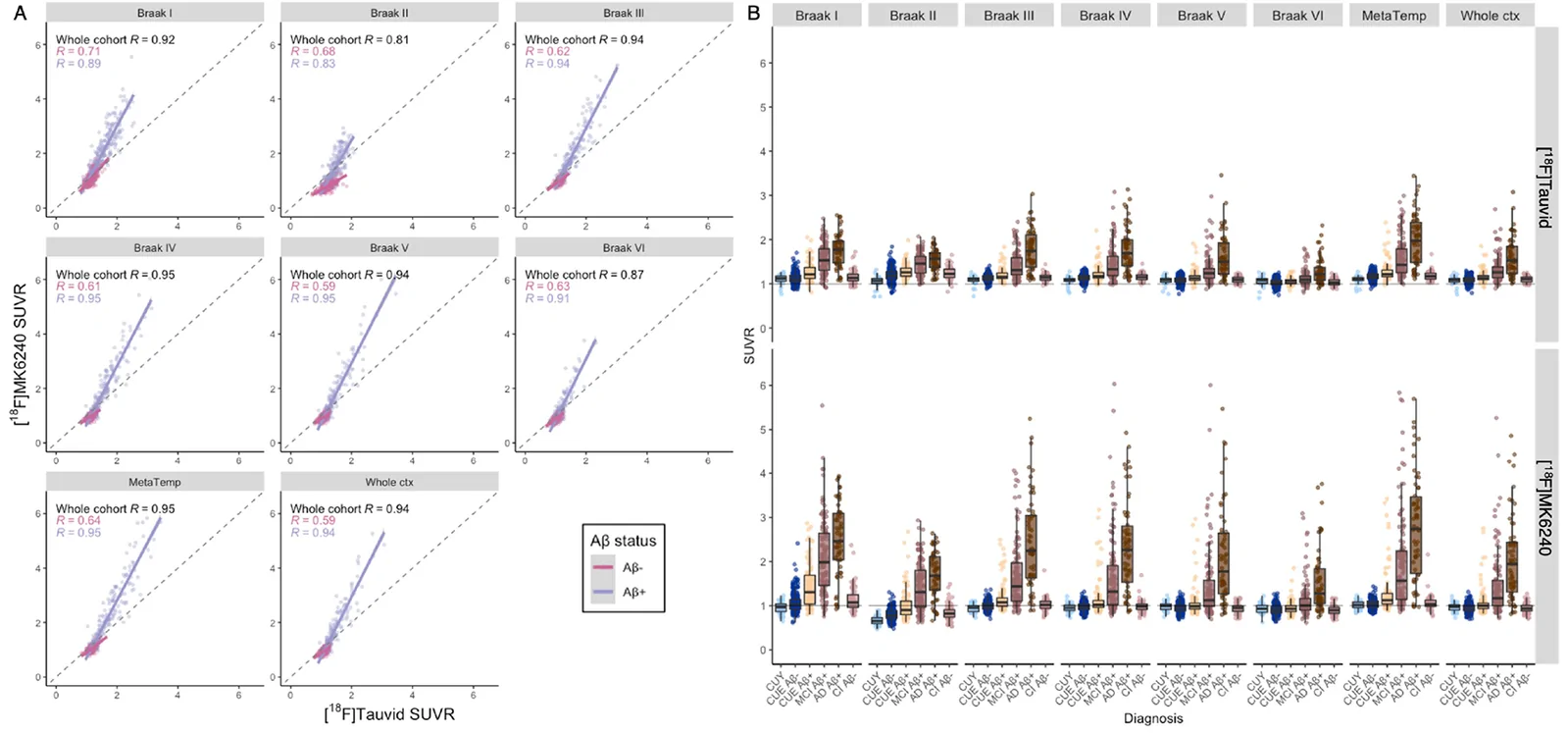
Associations between [^18^F]Tauvid and [^18^F]MK6240 in on-target regions Associations between [^18^F]Tauvid and [^18^F]MK6240 in on-target regions. A. Linear regressions. All associations were significant. B. Boxplots showing distribution within diagnostic group and Aβ status.

**Figure 3:**
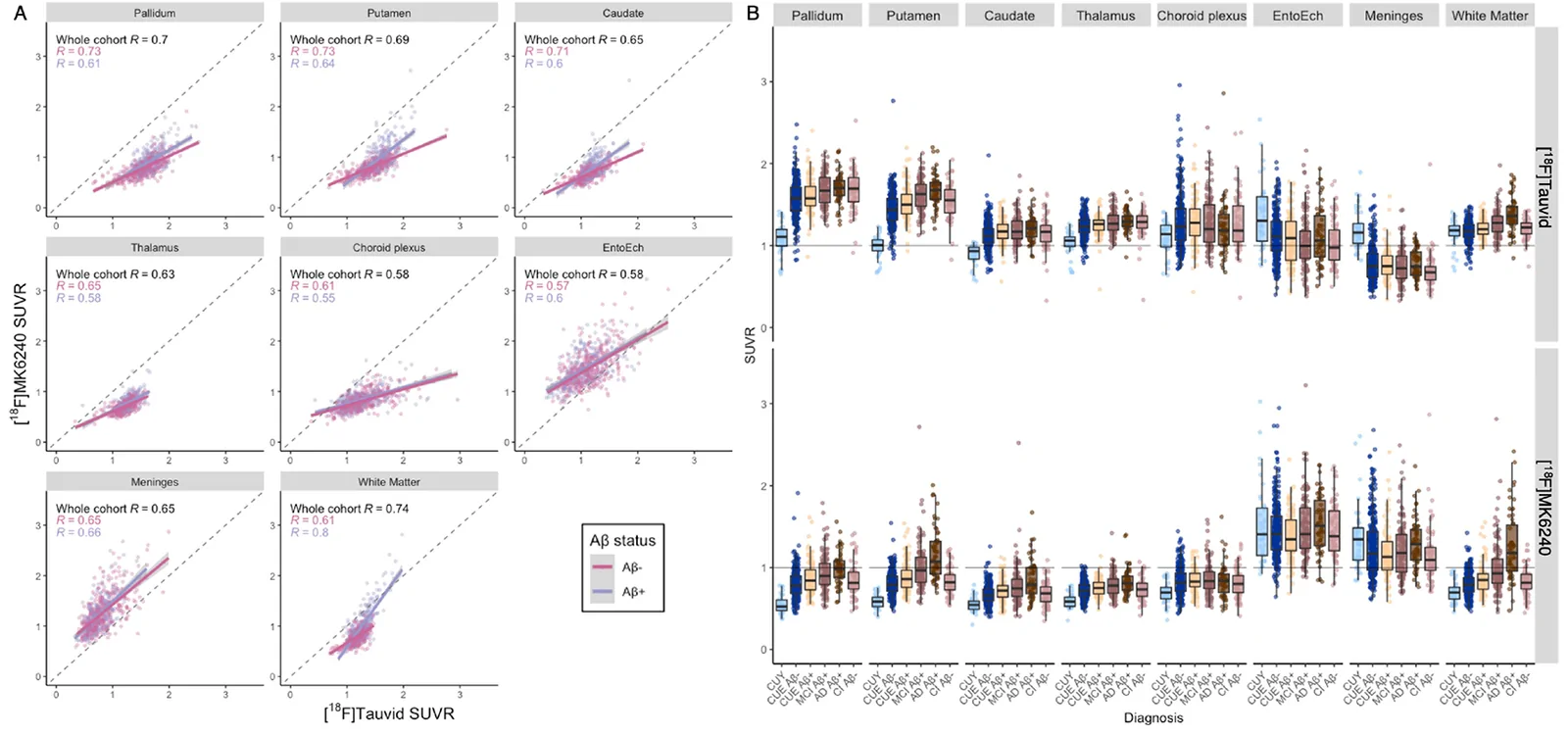
Associations between [^18^F]Tauvid and [^18^F]MK6240 in off-target regions Associations between [^18^F]Tauvid and [^18^F]MK6240 in off-target regions. A. Linear regressions. All associations were significant. B. Boxplots showing distribution within diagnostic group and Aβ status.

**Figure 4:**
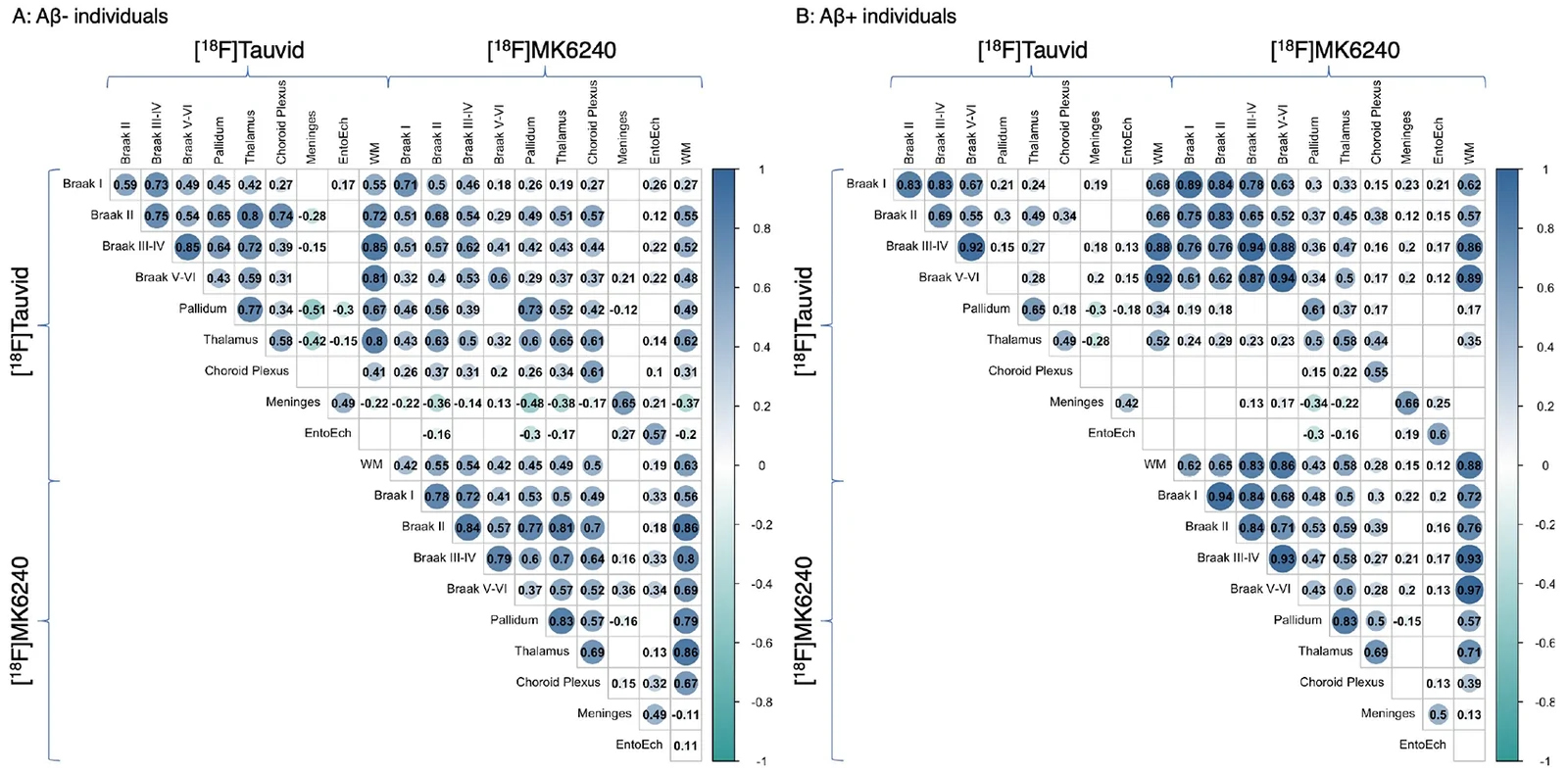
Associations within and between tracers in on and off-target regions Correlation matrices between on-target and off-target regions in A. Aβ− and B. Aβ+ individuals. All values shown were statistically significant.

**Figure 5:**
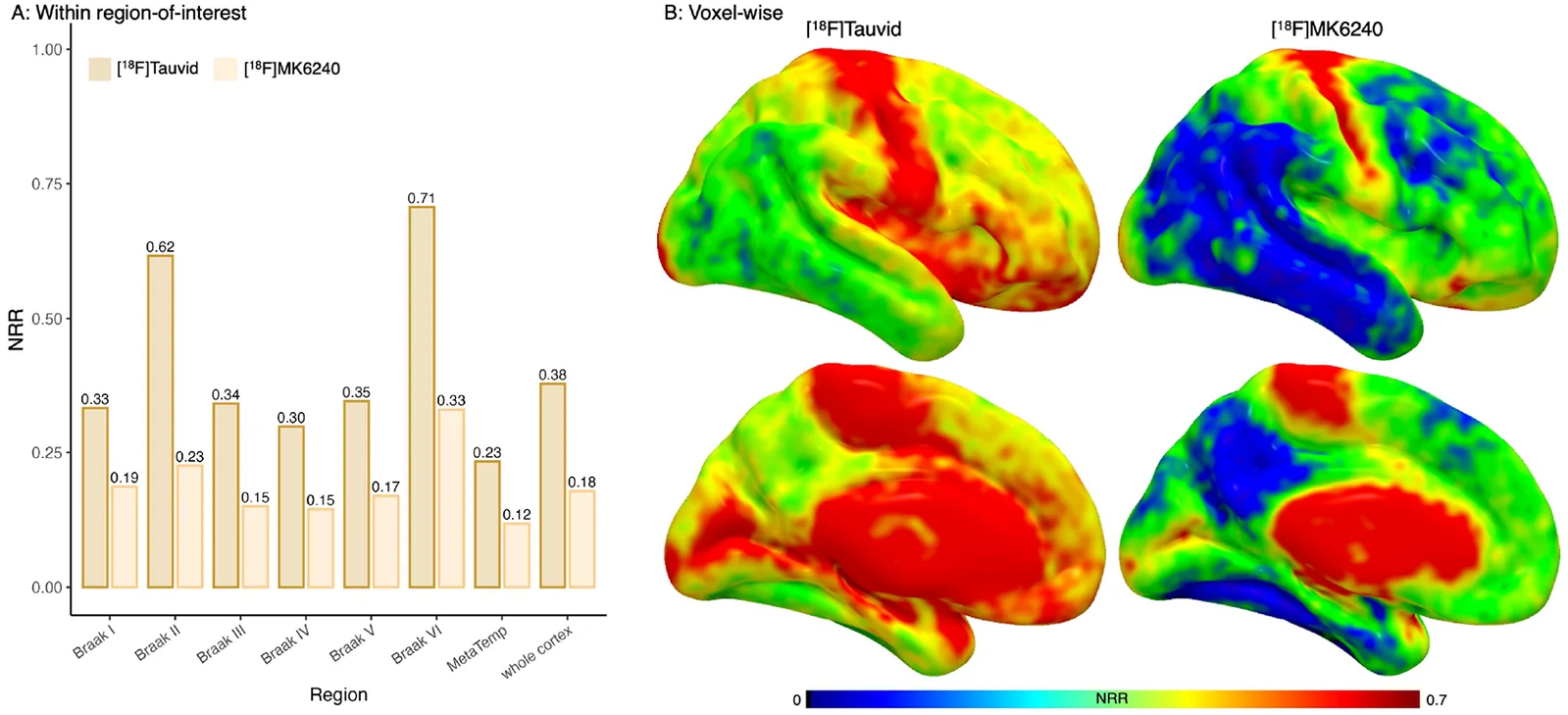
Noise-to-dynamic range ratio of tau-PET tracers Noise-to-dynamic range ratio of tau-PET tracers A. within ROI and B. voxel-wise.

**Table 1:** Demographics

Age (mean (SD))	CUY (N = 38)	CUO Aβ− (N = 293)	CUO Aβ+ (N = 97)	MCI Aβ+ (N = 123)	AD Aβ+ (N = 58)	CI Aβ− (N = 72)	*p*- value
	23.13 (1.9)	66.17 (8.0)	71.40 (7.1)	73.02 (7.4)	72.55 (8.0)	70.54 (8.7)	< 0.001
**Sex**							0.0107
Female	23 (60.5%)	169 (57.7%)	62 (63.9%)	62 (50.4%)	29 (50.0%)	27 (37.5%)	
Male	15 (39.5%)	124 (42.3%)	35 (36.1%)	61 (49.6%)	29 (50.0%)	45 (62.5%)	
**[^18^F]MK6240 meta-temporal SUVR (mean(SD))**	1.01 (0.1)	1.04 (0.1)	1.29 (0.5)	1.93 (1.1)	2.77 (1.2)	1.06 (0.2)	< 0.001
**[^18^F]Tauvid meta-temporal SUVR (mean(SD))**	1.10 (0.1)	1.17 (0.1)	1.28 (0.2)	1.57 (0.4)	1.99 (0.6)	1.18 (0.1)	< 0.001

## Data Availability

Data and materials used in this study are available from the corresponding author upon reasonable request. Requests are subject to institutional review to protect intellectual property and maintain confidentiality. Anonymized datasets can be shared with academic investigators for the purpose of replicating the study's results, contingent upon a signed data use or material transfer agreement.
